# Supplementary figures and images for: The Expression of Triticum aestivum Cysteine-Rich Receptor-like Protein Kinase Genes during Leaf Rust Fungal Infection
Source: Plants (Basel). 2023 Aug 14;12(16):2932. doi: 10.3390/plants12162932 (PMC10457733; doi:10.3390/plants12162932)

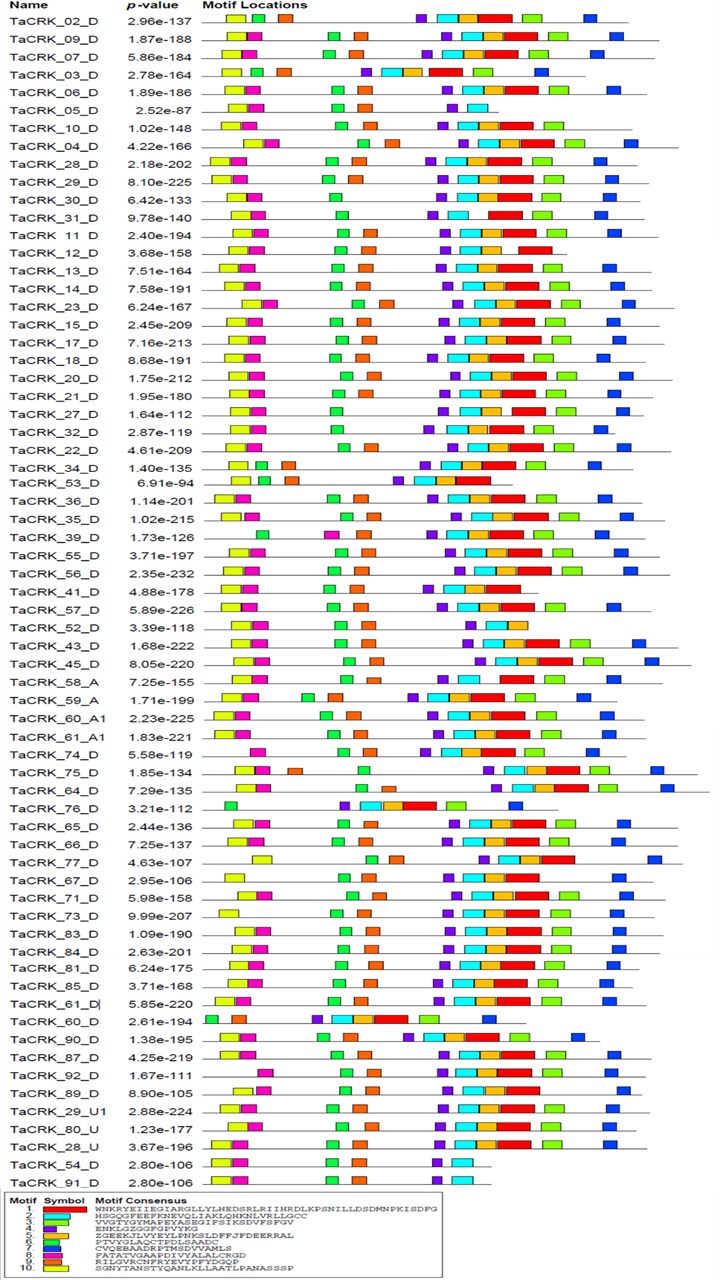

Supplement: Supplementary file 1 [file plants-12-02932-s001.zip › plants-2505459-supplementary/Supplements/Supplemental_Figure/Supplemental_Figure1.JPG]

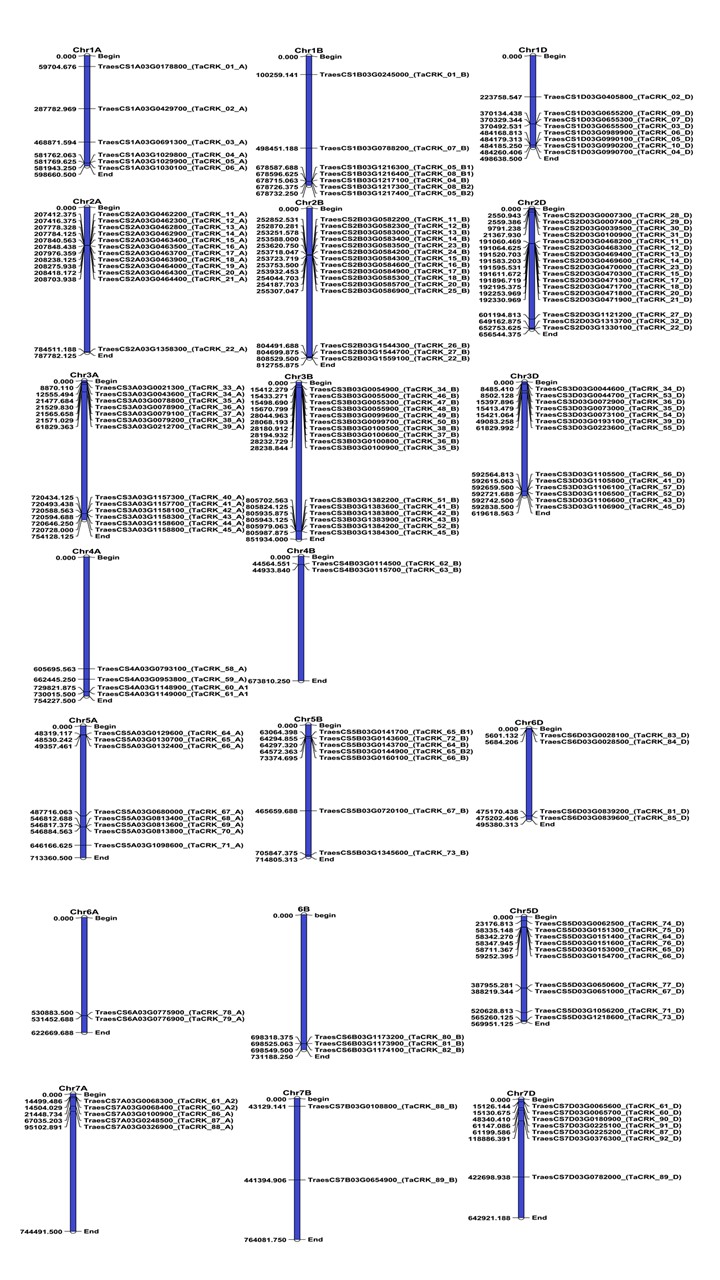

Supplement: Supplementary file 1 [file plants-12-02932-s001.zip › plants-2505459-supplementary/Supplements/Supplemental_Figure/Supplemental_Figure2.JPG]

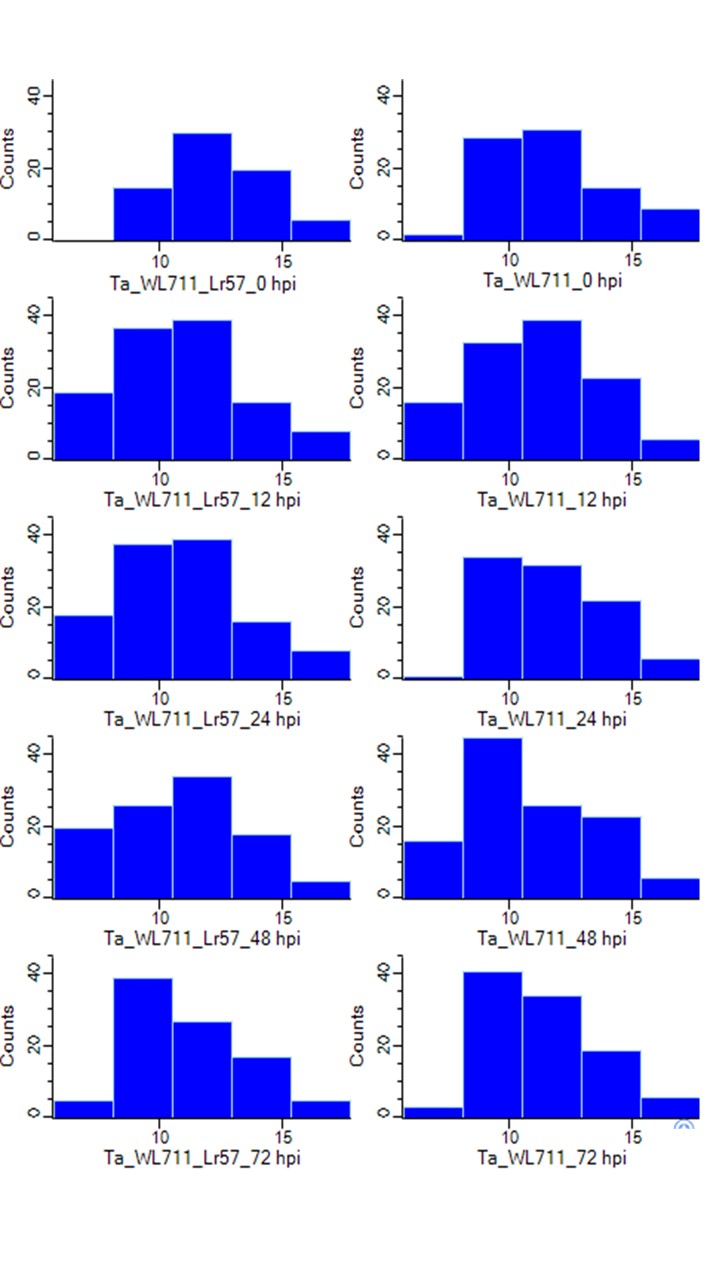

Supplement: Supplementary file 1 [file plants-12-02932-s001.zip › plants-2505459-supplementary/Supplements/Supplemental_Figure/Supplemental_Figure3.JPG]

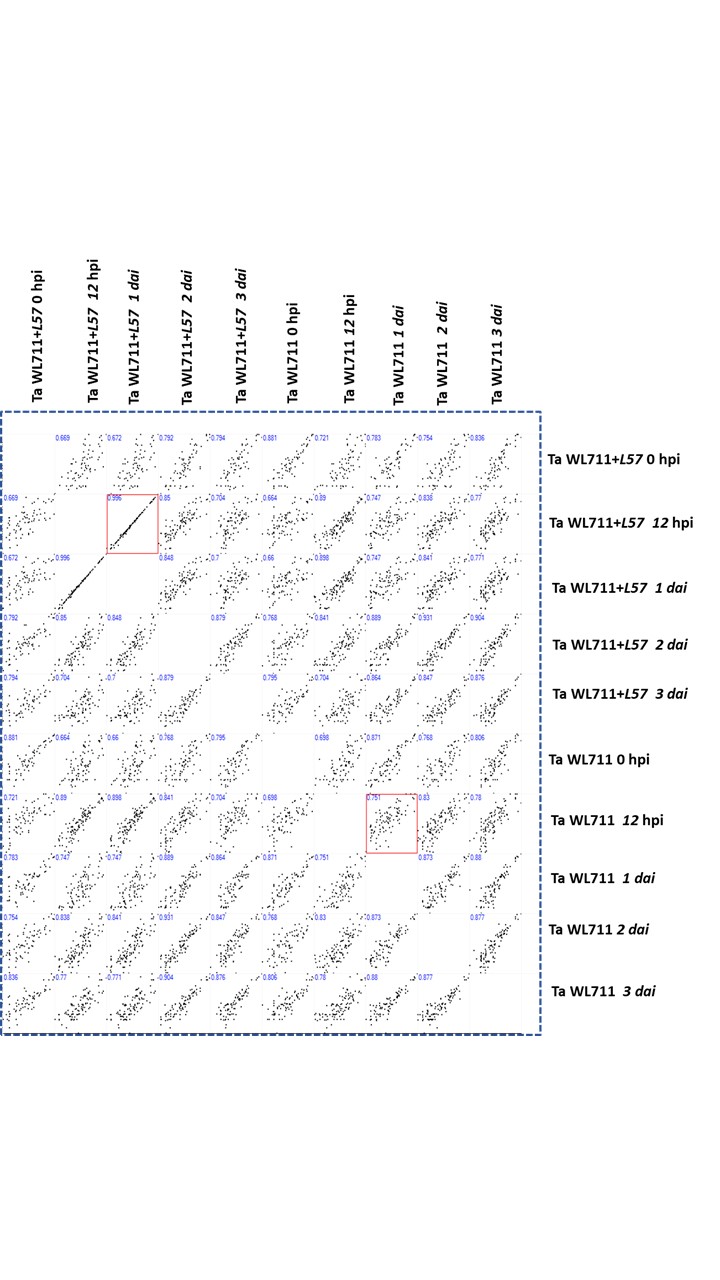

Supplement: Supplementary file 1 [file plants-12-02932-s001.zip › plants-2505459-supplementary/Supplements/Supplemental_Figure/Supplemental_Figure4.JPG]
